# Numerical Investigation of Deposition Characteristics of PLA on an ABS Plate Using a Material Extrusion Process

**DOI:** 10.3390/ma14123404

**Published:** 2021-06-19

**Authors:** Bih-Lii Chua, Sun-Ho Baek, Keun Park, Dong-Gyu Ahn

**Affiliations:** 1 Faculty of Engineering, Universiti Malaysia Sabah, Kota Kinabalu 88400, Malaysia; bihlii@ums.edu.my; 2 Department of Mechanical Engineering, Chosun University, Gwangju 61452, Korea; sunho123123@naver.com; 3 Department of Mechanical System Design Engineering, Seoul National University of Science and Technology, Seoul 01811, Korea; kpark@seoultech.ac.kr

**Keywords:** material extrusion, polylactic acid (PLA) filament, acrylonitrile butadiene styrene (ABS) plate, finite element analysis, deposition characteristics

## Abstract

Three-dimensional prototypes and final products are commonly fabricated using the material extrusion (ME) process in additive manufacturing applications. However, these prototypes and products are limited to a single material using the ME process due to technical challenges. Deposition of plastic on another dissimilar plastic substrate requires proper control of printing temperature during an ME process due to differences in melting temperatures of dissimilar plastics. In this paper, deposition of PLA filament on an ABS substrate during an ME process is investigated using finite element analysis. A heat transfer finite element (FE) model for the extrusion process is proposed to estimate the parameters of the ME machine for the formulation of a heat flux model. The effects of printing temperature and the stand-off distance on temperature distributions are investigated using the proposed FE model for the extrusion process. The heat flux model is implemented in a proposed heat transfer FE model of single bead deposition of PLA on an ABS plate. From this FE model of deposition, temperature histories during the ME deposition process are estimated. The results of temperature histories are compared with experiments. Using the calibrated FE model, a proper heating temperature of ABS for deposition of PLA is evaluated.

## 1. Introduction

The material extrusion (ME) process is a widely used additive manufacturing technique to create three-dimensional prototypes, and final products direct from a computer model. The material extrusion process, which is mainly popularized by the fused deposition modeling (FDM) process, is based on the extrusion of molten filament being deposited, cooled, and solidified in a layer-by-layer manner to form a three-dimensional part [1,2]. The two most commonly used materials in the ME process are amorphous polymers acrylonitrile butadiene styrene (ABS) and polylactic acid (PLA) [3,4,5,6,7]. The potential of application of the ME process has grown beyond prototyping and has been studied in several important industries such as automotive and aerospace, electronics, pharmaceutical, medical, dentistry, and water harvesting [8,9,10,11,12].

The ME process is depending on various process parameters such as layer height, print speed, extrusion rate, liquefier temperature, build plate temperature, size of nozzle, diameter of filament, and type of material [3,13]. The ME-fabricated prototype usually possesses poor surface roughness as compared to the stereolithography (SLA) process [14,15,16]. However, the ME process can produce desirable thermoplastics part that has higher strength. Application of different ME process parameters during the deposition process results in different mechanical properties of the final part [17,18,19,20]. 

Additively manufactured integrated devices such as pump, mixer, porous structures, electronics tongue, chemical reactants, electronics, and magnet sensor were investigated using the ME process by several researchers [21]. The integration of multi-functional components in a single part is achieved through the insertion of pre-fabricated components during printing, design of spatial orientation of a single material such as scaffolds and fluid channels, and/or application of spatial deposition of different materials at a specific surface or location [21,22,23]. Duarte et al. [24] developed an ABS microfluidic device with integrated sensing electrodes made of carbon nanotube-doped PLA using the ME process. However, the fusion between these two deposited materials was not evaluated.

In order to obtain the desired properties and functional materials for a ME process, several methods have been studied such as the inclusion of fillers into polymer matrix [22,25,26] and melt blending of different materials [7,27,28]. The inclusion of iron oxide and carbon nanotubes into polymers was reported to obtain desired magnetic and conductivity properties, respectively [20,22,24]. For tissue engineering, a blend of different polymers was used to increase hydrophilicity and improve cell adhesion and proliferation [23]. Studies of the ME process using fiber reinforced polymers [25,26] and blend polymers [7,28] at different blend ratios showed improvement in terms of mechanical properties.

Recently, Dhinesh et al. [7] proposed the concept of sandwiching PLA and ABS as alternate layers to improve the mechanical properties of fabricated parts via the ME process. The PLA/ABS sandwich concept has produced specimens with lesser strain to stress ratio than pure ABS specimens. The ability to print two dissimilar materials as a single part improves the potential of the ME process to tailor the properties and functionalities according to different applications. The challenge is to ensure inter-layer bonding is properly formed between the materials of different thermal conductivities by heating the polymer surface above its sintering temperature [20]. In order to obtain suitable ME printing parameters, the fusion characteristics between two dissimilar thermoplastics, which rely on thermal to form reaction bonding, should be investigated. In view of expanding combinations of materials with different mechanical properties, the ME process can grow with proper understanding of the deposition process via numerical investigation.

Finite element analyses (FEAs) have been applied to investigate a ME process. Zhang and Chou [29] demonstrated that the FDM process can be simulated using finite element analysis (FEA) to investigate the effect of tool-path on the formation of stress and distortion patterns induced in part. Transient heat transfer models were proposed to estimate the temperature of the ME process and the formation of bonds for a single filament deposition process [30,31]. However, their models are limited to the formation of a bond between two adjacent depositions of the same material. The effect of the heated build plate, which is typically applied to improve adhesion and avoid warping of the printed part, is not considered in their analysis. Applications of finite element analyses for the ME process are significantly less than those for metal additive manufacturing. Heat transfer finite element analysis can be developed to improve the design of process parameters on the deposition characteristics of an additive manufacturing process [32]. Proper deposition characteristics using the two common materials ABS and PLA via ME process to form a single part is yet to be investigated. With the growing need to form functional parts, proper bonding between these dissimilar materials through the selection of process parameters should be ensured.

In this paper, an ABS substrate is applied as the build plate add-on and a PLA filament is used as the deposited material for the experiments and finite element analyses of the material extrusion process. The effects of printing temperature and stand-off distance on temperature distributions at the nozzle and extruded material are investigated experimentally and numerically to estimate the parameters for the ME machine MakerBot Replicator. A transient heat transfer finite element (FE) model for the deposition process is proposed and calibrated to investigate the temperature histories during the ME deposition process based on an algorithm proposed for directed energy deposition (DED) in [32]. Finally, heat transfer finite element analyses (FEAs) using the calibrated FE model are performed to evaluate the proper plate heating temperature of the ABS for deposition of PLA.

## 2. Finite Element Analysis and Experiments of Material Extrusion Process

### 2.1. Material Extrusion Process of Extruder Unit

**Figure 1:**
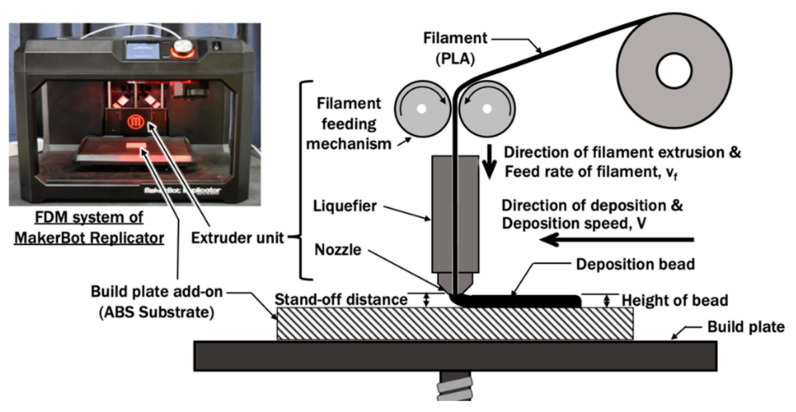
Schematic diagram of a material extrusion process.

The material extrusion process applied in this paper is based on the fused deposition modeling (FDM) system of MakerBot Replicator (5th generation) [33]. The schematic of the ME process is illustrated in Figure 1.

A PLA filament (Supplier: Makerbot; Color: True White) is fed into an extruder unit and melted by the liquefier with a maximum liquefier temperature capacity of 256 °C. The melted filament is extruded out through a 0.4 mm nozzle to form a deposition bead on the substrate. The diameter of the PLA filament is 1.75 mm. The deposition speed V is set at 10 mm/s. A 40 mm straight bead is deposited during the experimental study. The height of bead h_b_, which defines the layer height, is controlled by the stand-off distance between the nozzle and substrate. The height of the bead is usually equal to the stand-off distance for a ME process. In this paper, an 2.5 mm thick ABS substrate (Supplier: Oyang Inc. Korea; Color: White) is applied as the build plate add-on such that the deposition experiment of PLA on the ABS plate in Section 2.2 can be conducted and analyzed.

The FDM system using Makerbot Replicators is a propriety system. In order to analyze the ME process using this FDM system, the feed rate of filament v_f_ is estimated at 7.7436 mm/s from a deposition experiment. The liquefier temperature T_L_ is controlled automatically based on set target printing temperature T. In order to obtain the heat input at the liquefier for the numerical investigation of this material extrusion process, a finite element model of liquefier and nozzle is proposed in this section and results of analyses are compared with actual measured temperature T_n,actual_ at the nozzle.

#### 2.1.1. Finite Element Model for Extrusion Process of Filament

**Figure 2:**
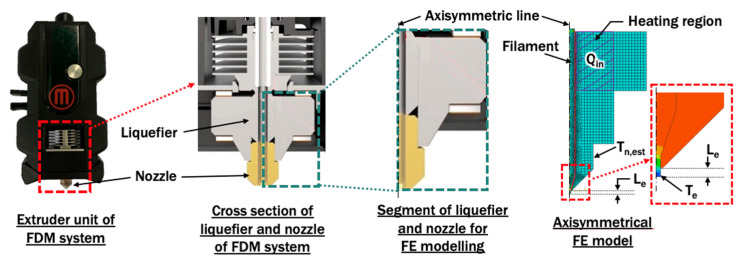
Development of axisymmetrical finite element model from actual extruder unit of FDM.

An axisymmetrical FE model for liquefier and nozzle is proposed based on the actual construction of an extruder unit, as shown in Figure 2. Steady state heat transfer analyses are performed using a commercial code Abaqus V6.12. In this FDM system, heat input Q_in_ of the heating region of liquefier should be estimated as in Equation (1):

Q_in_ = π ρ d^2^ v_f_ C_p_ (T_p_ − T_∞_)/4, (1)

where ρ, d, C_p_, T_p_ and T_∞_ are density of filament, diameter of filament, the specific heat of filament at room temperature, preset printing temperature, and room temperature of 20 °C, respectively.

The material applied for filament is PLA. Brass is set as the material for liquefier and nozzle in the proposed FE model. A natural convection of 5 W/m^2^·K with an ambient temperature of 20 °C is applied at the outer boundary of the extruder unit which is in contact with air [34]. Table 1 summarizes the variations of printing temperature and extruded length of filament from the end of the nozzle orifice, that have been applied in the axisymmetrical finite element model. The estimated temperature at nozzle T_n,est_ and temperature at end of extruded material T_e_ are observed and compared with validation and verification experiments, respectively. The contact interface between PLA and the extruder unit is assumed to be perfectly conductive. 

#### 2.1.2. Material Properties

**Figure 3:**
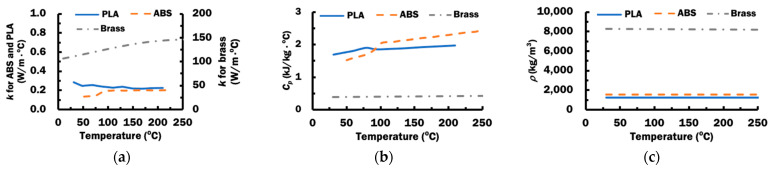
Temperature-dependent material properties for ABS, PLA, and brass: (**a**) thermal conductivities, *k*; (**b**) specific heat capacities, *C_p_*, (**c**) densities, *ρ*.

Temperature-dependent thermal conductivities, specific heat capacities, and densities for PLA, ABS, and brass, as shown in Figure 3, are applied in the finite element model [35,36]. The glass transition temperatures of ABS and PLA are 110–120 °C and 60–65 °C, respectively [6,37].

#### 2.1.3. Validation of Finite Element Model Using Temperature Measurement at Nozzle

**Figure 4:**
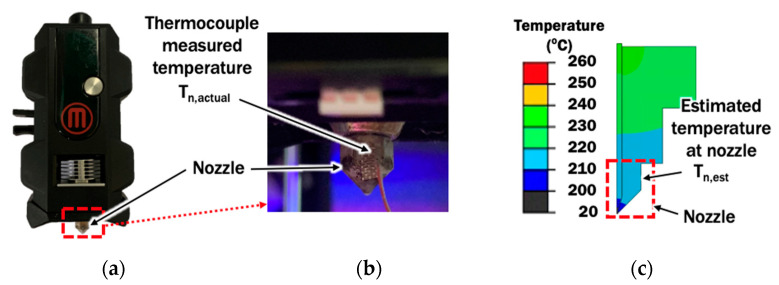
Temperature measurement at nozzle: (**a**) Location of nozzle at the extruder unit; (**b**) Location of the thermocouple at nozzle; (**c**) Estimated temperature at the corresponding location of thermocouple in FE model.

In order to validate the proposed FE model for liquefier and nozzle, a J-type thermocouple (Omega fine wire thermocouple) is installed at the vertical side of the nozzle, as shown in Figure 4. The temperature at nozzle T_n,actual_ during a material extrusion process is acquired using a standalone multi-channel data logger (Graphtec midi logger GL240). The temperature measured is compared with the temperature estimated at the corresponding location in the FE model.

#### 2.1.4. Verification of Proper Extruded Length Using Temperature Measurement at Deposit-Substrate Interface (DSI) during Deposition Process

**Figure 5:**
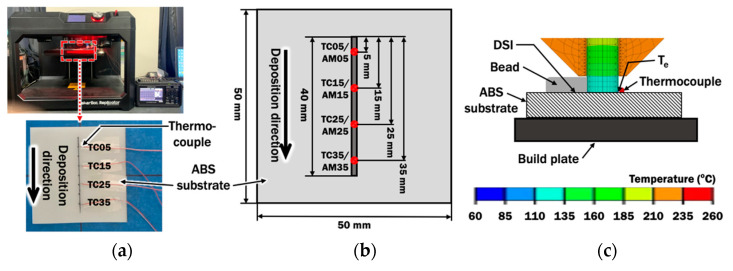
Temperature measurement at deposit–substrate interface (DSI) during deposition: (**a**) Locations of thermocouples at substrate during material deposition; (**b**) Corresponding locations of thermocouples at FE model; (**c**) Estimated temperature at end of extruded material that is in contact with thermocouples and ABS substrate during deposition.

In order to verify the selected extruded length based on results of temperatures at end of extruded material T_e_ from the FE model for liquefier and nozzle, J-type thermocouples (Omega fine wire thermocouple) are installed on the build plate add-on, as shown in Figure 5a. The measured peak temperatures at different locations are acquired and averaged as T_deposit_ during a material extrusion process. Figure 5b illustrates the correlations between locations of thermocouples TC05, TC15, TC25, and TC35 and locations of AM05, AM15, AM25, and AM35 in the FE model, respectively. The mean peak temperature T_deposit_ is compared with the estimated temperature at end of extruded material T_e_ in the selected FE analysis, as they are connected at deposit-substrate interface DSI during the deposition process in Figure 5c. The selected extruded length will be used to determine the suitable standoff distance between the nozzle and substrate.

### 2.2. Deposition Process of PLA on ABS Plate

This section describes the setup of an FE model to simulate the deposition process of PLA filament on an ABS plate. A 40 mm PLA straight bead is deposited at deposition speed V of 10 mm on a 2.5 mm thick ABS substrate during the material deposition process.

#### 2.2.1. Finite Element Model for Deposition Process

**Figure 6:**
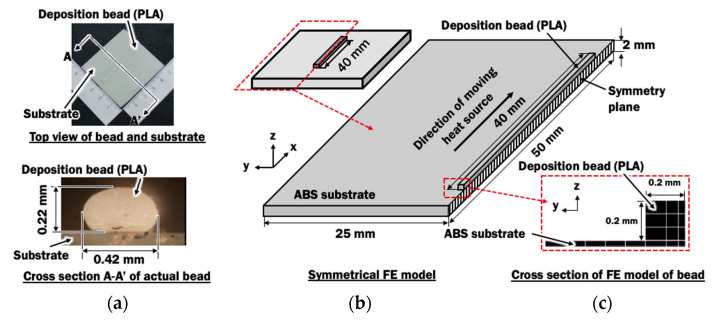
(**a**) Dimension of actual bead; (**b**) A symmetrical finite element model for deposition process. (**c**) Cross section of FE model of bead.

In order to evaluate the distributions of temperature during deposition of PLA material on a dissimilar material of ABS substrate, a transient heat transfer finite element model with an element activation algorithm proposed in [32] is required. Figure 6 illustrates the three-dimensional symmetrical FE model with one straight bead lying in the middle of a 50 mm × 50 mm substrate. A rectangular bead is estimated for the FE model from the cross section of the actual bead deposited using an ME process with stand-off distance of 0.2 mm. The height of bead h_b_ is assumed to be the stand-off distance between the nozzle and substrate. The FE analyses of single bead deposition are conducted using parameters shown in Table 2.

#### 2.2.2. Heat Source Model

**Figure 7:**
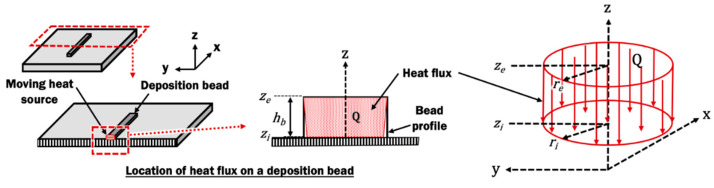
Volumetric top-hat distribution of heat flux with penetration depth model on a deposited bead.

The extruded material from the nozzle contains heat. The heated material is constantly deposited throughout the material extrusion process, resembling a heat source acting on the deposited material. In the simulation of the material extrusion process, a moving heat source with a volumetric top-hat distribution of heat flux *Q* with penetration depth through the height of bead h_b_ is applied as heat input, as described by mathematical models in Equations (2) and (3):

(2) $$Q\left(r,Z\right)=\{\begin{array}{cc} \frac{3\eta Q_{in}}{\pi h_{b}\left(r_{i}{}^{2}+r_{e}r_{i}+r_{e}{}^{2}\right)} & ,\;\;\;\;\;\;\;\;\;\;\;r\leq r_{0}\;\mathrm{or}\;z_{i}\leq Z\leq z_{e}\\ 0 & \;,\;\;\;r>r_{0}\;or\;z_{i}>Z\;\mathrm{or}\;Z>z_{e} \end{array}$$

(3) $$r_{o}\left(Z\right)=r_{e}+\left(\frac{r_{i}-r_{e}}{z_{i}-z_{e}}\right)\left(Z-z_{e}\right)$$

where *Q*_in_ is the heat input of the heating region of liquefier, *r* is the radial distance from the center of heat source, *Z* is the z-coordinate relative to the frame of heat source, *η* is the efficiency, *r_e_* is the effective heat source radius at the surface of the bead, *r_i_* is the effective heat source radius at distance *d_p_* from the surface of the bead, *z_e_* is the z-coordinate at the surface of the bead, *z_i_* is the z-coordinate at distance *d_p_* from the surface of the bead, and *r_o_* is the effective radius at arbitrary z-coordinate. The parameters applied in the FE model is summarized in Table 3. The *r_i_* is assumed to be slightly smaller than *r_e_* because the temperature of deposited material near the substrate is lower than that measured near the nozzle. The volumentric top-hat distribution of heat flux is illustrated in Figure 7.

The efficiency of the heat flux model has been calibrated according to the temperature measured at DSI during an experiment with a printing temperature of 230 °C. This calibration is necessary because heat loss may start to occur when melted PLA in the liquefier is extruded through the nozzle before it is being deposited. From the calibration, 100% efficiency is found suitable due to negligible heat loss along the short distance between the liquefier and the ABS substrate. The calibrated efficiency is applied for all deposition processes using different printing temperatures, as summarized in Table 4.

#### 2.2.3. Numerical Investigation of Deposition Process with Plate Heating

The finite element model in the Section 2.2.1 is applied to investigate the influence of plate heating on deposition characteristics of PLA on ABS. In these analyses, the ABS substrate is acted as the plate with heating. The heating of ABS substrate is considered because it has a higher melting point and glass transition temperature as compared to PLA [6]. In this paper, the glass transition temperatures (T_g_) for ABS and PLA are assumed to be 110 °C and 65 °C, respectively [6,37]. Therefore, the deposited PLA material must raise the temperature of ABS above 110 °C for the fusion between ABS and PLA to occur. The combinations of process parameters between ABS substrate heating temperature and printing temperature, as in Table 5, are applied to the proposed FE model for investigation. The ABS initial temperature is assumed to be equal to the ABS heating temperature.

## 3. Results and Discussion

### 3.1. Effects of Printing Temperature and Stand-Off Distance on Temperature Distributions of Extruder Unit

Effects of printing temperature and stand-off distance on temperature distributions of extruder unit during the extrusion process of filament are investigated using results of heat transfer finite element analyses (FEAs). The proposed axisymmetrical FE model for liquefier and nozzle is applied. The FE model is validated and discussed by comparing the results of FEAs with actual temperature measurements in the following sections.

#### 3.1.1. Validation of FE Model for Extrusion Process

**Figure 8:**
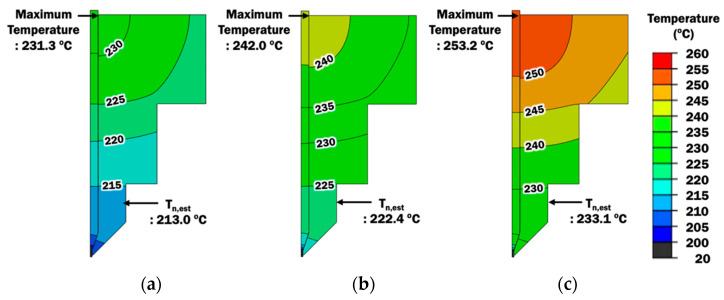
Temperature distribution at extruder unit for different printing temperatures: (**a**) T_p_ = 230 °C; (**b**) T_p_ = 240 °C; (**c**) T_p_ = 250 °C.

Figure 8 shows temperature distributions at the extruder unit for different printing temperatures. The liquefier, nozzle, and PLA filament are the only components in the extruder unit that are considered in the proposed FE model for the extrusion process. This is because the main heat transfer phenomenon during the extrusion process occurs within these components. The heat input in the FE model is estimated based on the printing temperature, which is controlled by the ME system. Maximum temperatures of the FE model are found at the liquefier and are slightly higher than the desired printing temperature, in the range not more than 3.2 °C. This indicates that the heat input has been estimated properly.

In order to further validate the FE model, the temperature is measured at the nozzle during the material extrusion process. Estimated temperatures from the results of FEAs are taken at the corresponding location in the FE model. Table 6 shows the measured temperatures and estimated temperatures at the nozzle. Both measured and estimated temperatures show good agreement with small temperature differences in the range not more than 2 °C. Hence, the FE model for the extrusion process is validated and its estimated temperature distributions can be used to investigate the effects of printing temperature and stand-off distance on temperature distributions.

#### 3.1.2. Effects of Printing Temperature and Extruded Length of PLA Filament on Temperature Distributions and Selection of Stand-Off Distance

**Figure 9:**
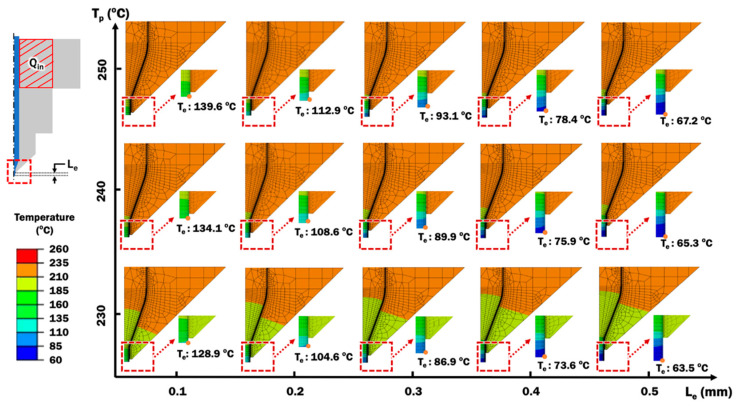
Estimated temperature distributions of PLA material from heat transfer analysis using different combinations of printing temperatures and extruded length of filament from the end of the nozzle orifice.

Heat transfer FEAs are performed using the validated FE model of extruder unit for different combinations of printing temperature and extruded length of PLA filament. Temperature distributions for different combinations of printing temperature and extruded length of PLA filament are observed, as shown in Figure 9. From the result of heat transfer FEA in Figure 9, temperatures at the end of extruded material, T_e_, are estimated.

**Figure 10:**
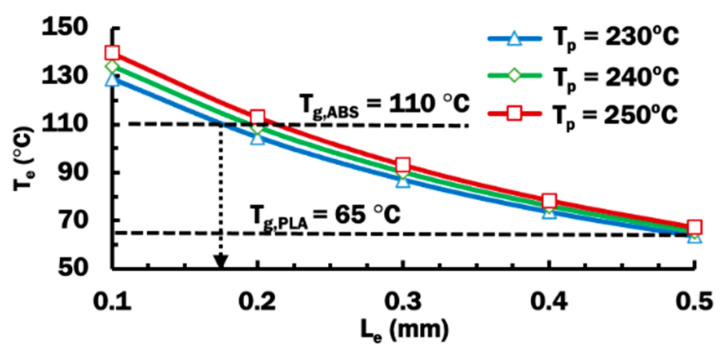
Influence of extruded length of PLA filament on the temperature at end of extruded material, T_e_, using different printing temperatures.

Figure 10 shows the influence of the extruded length of PLA filament on the temperature at end of extruded material, T_e_, using different printing temperatures. The temperature at end of extruded material reduces when the extruded length increased for the same printing temperature of 230 °C. For other higher printing temperatures, similar declining trends of temperature T_e_ according to longer extruded lengths are observed. This is due to the fact that a longer extruded length of PLA filament from the nozzle orifice has a larger surface in contact with air for convective cooling. Higher printing temperature applied to the system results in a hotter temperature at end of extruded material. This is consistent with greater heat input into the FE model during the extrusion process, as a result of higher printing temperature.

In order for fusion between ABS and PLA to occur, the temperature of deposited PLA material must be higher than the glass transition temperature of ABS, T_g,ABS_. When the amorphous ABS polymer is heated over its T_g,ABS_, the polymer structure turns into a rubbery state [37]. The rubbery state of ABS substrate allows its polymer chain to move and mix with the deposited PLA materials to form a strong adhesive bond [38]. Figure 10 shows that all three printing temperature cases of extruded length at 0.175 mm or less have their T_e_ of at least 110 °C. In the case of 0.2 mm extruded length, a printing temperature of 250 °C is required for its T_e_ to be greater than 110 °C. For those cases that have T_e_ less than 110 °C, a weak or no bonding may be formed between deposited PLA and ABS substrate. The stand-off distance can be estimated to be the extruded length, as illustrated in Figure 5c. Therefore, a proper stand-off distance can be selected from these cases that produce T_e_ to be greater than 110 °C. In this paper, a stand-off distance of 0.2 mm is selected with the knowledge that the utilized ME system is designed to work at a default stand-off distance of 0.2 mm [33].

#### 3.1.3. Relationship Between Estimated Temperature at End of Extruded Material and Measured Temperature at DSI during Deposition Process

**Figure 11:**
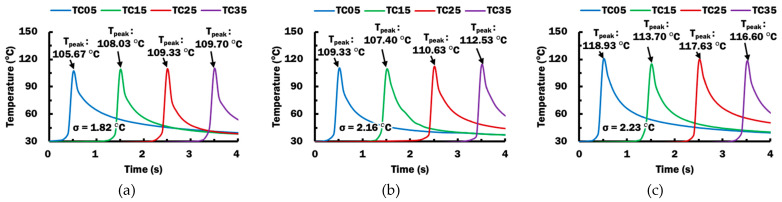
Temperature histories and peak temperatures measured at TC05, TC15, TC25, and TC 35 during the deposition process using different printing temperatures: (**a**) T_p_ = 230 °C; (**b**) T_p_ = 240 °C; (**c**) T_p_ = 250 °C.

The deposition experiment described in Section 2.1.4 is set up and performed using the selected stand-off distance of 0.2 mm. Figure 11 shows the temperature histories measured by thermocouples TC05, TC15, TC25, and TC 35 during the deposition process for different printing temperatures. The temperature for each thermocouple reaches the peak temperature T_peak_ when extruded material is deposited on top of the corresponding thermocouple. For a material extrusion process, peak temperatures recorded for all thermocouples are almost similar with standard deviation, σ, not more than 2.23 °C. Hence, deposition temperatures throughout the length of deposition bead at the deposit–substrate interface, DSI, are deduced to be similar. The measured peak temperatures for all thermocouples are averaged as T_deposit_ during a material extrusion process, as summarized in Table 7.

Estimated temperatures at end of extruded material T_e_ for selected L_e_ at 0.2 mm are obtained from results of FEAs in Figure 9 and tabulated in Table 7 for comparison. The mean peak temperature T_deposit_ is found to be slightly higher than the estimated temperature at end of extruded material T_e_ in the selected FEAs. The differences between T_deposit_ and T_e_ are not more than 3.82 °C. These small differences indicate that the T_e_ can be used to predict the temperature at DSI during the ME deposition process. Based on this relationship, the proper extruded length and stand-off distance for the proper deposition process can be investigated and verified from the viewpoint of estimated temperature at end of extruded material T_e_.

### 3.2. Effects of Printing Temperature on the Temperature Histories during Deposition Process

**Figure 12:**
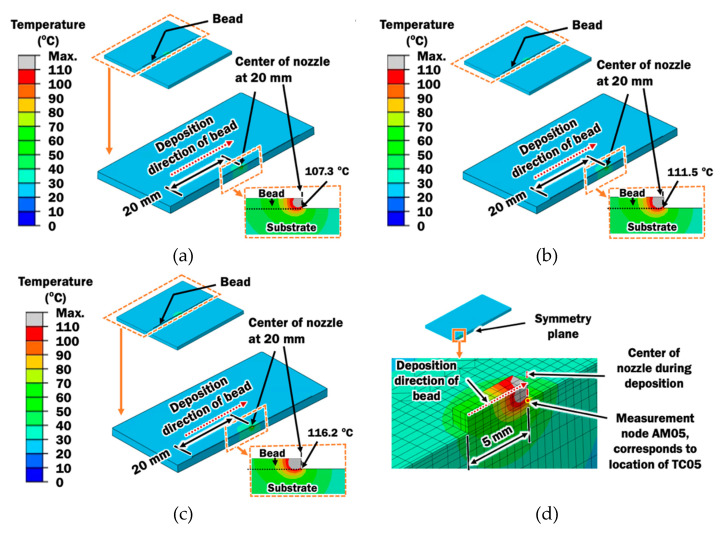
Estimated temperature distributions during the deposition of a bead with nozzle’s location at 20 mm from the start point for different printing temperatures: (**a**) T_p_ = 230 °C; (**b**) T_p_ = 240 °C; (**c**) T_p_ = 250 °C; (**d**) location of measurement node AM05 on the ABS substrate corresponding to locations of thermocouples TC05.

In order to investigate the effect of printing temperature on temperature histories, transient heat transfer finite element analyses are performed on the proposed FE model in Section 2.2 to simulate the heat transfer phenomena during the ME deposition process using the selected stand-off distance of 0.2 mm. The deposition of heated PLA material to form a straight bead is simulated with an element activation algorithm and a calibrated moving heat source model. Figure 12 exhibits estimated temperature distributions during the deposition of a bead with nozzle’s location at 20 mm from the start point for different printing temperatures. The temperature histories at different measurement nodes on the FE model, as illustrated in Figure 12d can be estimated.

**Figure 13:**
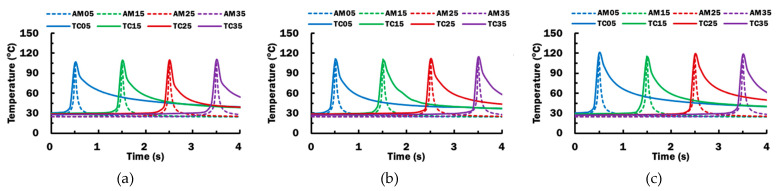
Temperature histories measured at locations of thermocouples TC05, TC15, TC25, and TC35 during the end of deposition from heat transfer FEAs for different printing temperatures: (**a**) T_p_ = 230 °C; (**b**) T_p_ = 240 °C; (**c**) T_p_ = 250 °C.

Validation of the proposed FE model for the deposition process is required for the numerical investigation to be useful. The temperature histories at AM05, AM15, AM25, and AM35 on the FE model are compared with those measured by TC05, TC15, TC25, and TC35, respectively in Figure 13. It reveals that both estimated and measured temperature histories has their temperature peak at a similar time and similar value. However, it shows that thermocouples cool slower than estimated after the peak temperature. This could be justified such that the heat does not transfer perfectly to ABS substrate in actual condition. It cools slowly because the thermocouple wire laid on the ABS substrate has prevented the deposited PLA to contact perfectly with the ABS substrate for the cooling heat transfer. Furthermore, the contact area in the FE model is larger due to the assumption of the rectangular bead. These discrepancies between the FE model and actual condition result in the mismatch of temperature histories after reaching peak temperature.

Nevertheless, it shows good agreement between actual measurement and estimated in FEAs on the heating characteristics and peak temperatures at the time it is deposited and in contact with the substrate. Good agreement on the heating characteristics and peak temperatures are more important for the investigation of deposition characteristics of PLA on an ABS plate. The cooling characteristics during deposition, which can be calibrated by adjustment of coefficient of convection, are not required for any of the discussions in this paper. Therefore, the FE model is validated.

**Figure 14:**
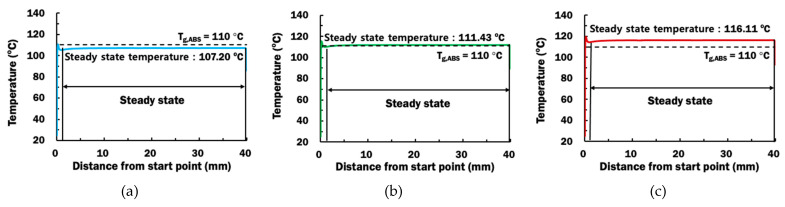
Estimated peak temperatures along the length of bead from start point for different printing temperatures: (**a**) T_p_ = 230 °C; (**b**) T_p_ = 240 °C; (**c**) T_p_ = 250 °C.

Transient heat transfer FEAs provide thorough information pertaining to the deposition temperature at DSI during the ME deposition process. The deposition temperature is estimated as the peak temperature obtained from nodes along the DSI of the straight bead. Figure 14 shows the estimated peak temperatures along the length of straight bead for different printing temperatures. It is revealed that the deposition temperature reaches a steady state deposition temperature after 2 mm of deposition.

From Figure 14, steady state deposition temperatures are estimated to be 107.20 °C, 111.43 °C, and 116.11 °C for printing temperatures of 230 °C, 240 °C, and 250 °C, respectively. The steady state deposition temperature increases with increasing printing temperature, which is consistent with the increase of heat input. Glass transition temperature of ABS is used to predict the rubbery region at the ABS substrate and suitability of the ME process for depositing PLA material on ABS substrate. From the steady state temperatures, it is predicted that deposition processes using printing temperatures of 240 °C and 250 °C are suitable for depositing PLA materials on ABS substrate due to their deposition temperatures are greater than the glass transition temperature of ABS.

### 3.3. Effects of Plate Heating Temperature on the Temperature Distributions of Deposited Bead and Substrate

**Figure 15:**
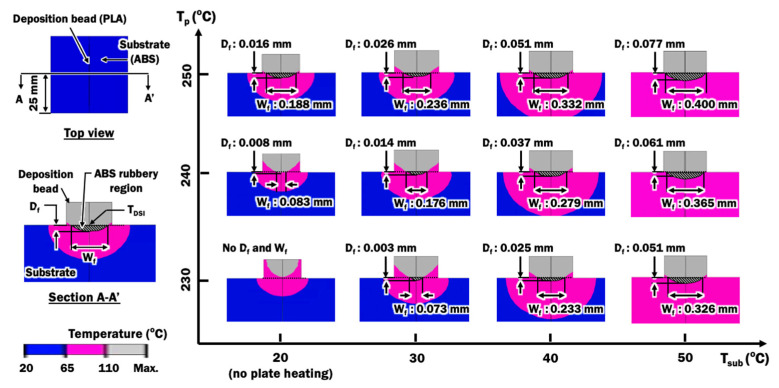
Estimated temperature distributions of PLA material from heat transfer analysis using different combinations of printing temperatures and extruded length of filament from the end of the nozzle orifice.

**Figure 16:**
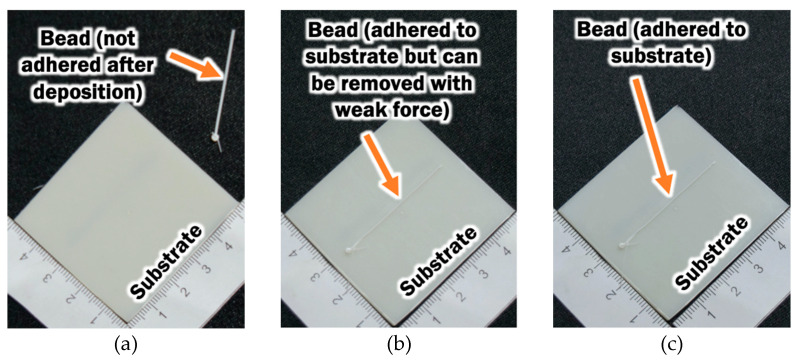
Bead deposited on substrate after ME process using different printing temperature and no plate heating: (**a**) T_p_ = 230 °C; (**b**) T_p_ = 240 °C; (**c**) T_p_ = 250 °C.

Although the steady state deposition temperature provides vital information to determine the suitability of the ME process for depositing PLA material on ABS substrate, the region on the ABS substrate that has been turned into the rubbery state is not properly known. Temperature distributions of the cross section of the bead and substrate at the middle of the substrate are estimated from results of FEAs in order to investigate the size of the rubbery region in the ABS substrate during deposition, as illustrated by the hatch region in Figure 15. The size of the rubbery region is quantified by the fused contact width W_f_ and depth D_f_. From the temperature distributions of FEAs in Section 3.2, rubbery regions are estimated for deposition processes without plate heating. In this case, the initial temperature of the substrate T_sub_ is set as room temperature of 20 °C. From the fused contact width, it reveals that deposition processes with printing temperatures of 230 °C and 240 °C are indeed not suitable for depositing PLA materials on ABS substrate. This is due to the fact that the W_f_ is too small (less than 0.01 mm) for the ABS and PLA to have sufficient fusion bonds. This is supported by the observations of bead adhesion on the substrate after the ME process, as shown in Figure 16.

**Figure 17:**
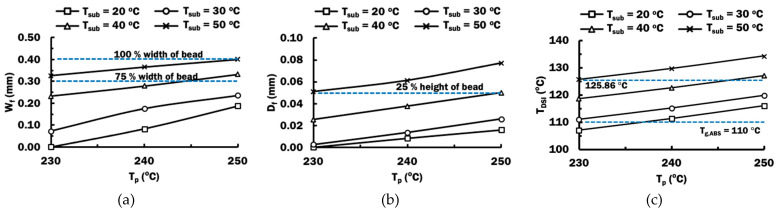
Estimated parameters from heat transfer FEAs for different combinations of printing temperatures and plate heating temperature: (**a**) Fused contact width W_f_; (**b**) Depth of rubbery region D_f_; (**c**) Deposition temperature at DSI T_DSI_.

Plate heating is considered to improve the fusion contact area between ABS and PLA. Transient heat transfer FEAs using parameters in Table 5 are conducted to investigate the effects of plate heating temperature on the formation of the rubbery region in the ABS substrate during deposition, as shown in Figure 15. The deposition temperature T_DSI_ is estimated for different combinations of printing temperatures and plate heating temperatures. In these cases, the initial temperature of the substrate T_sub_ is set equal to the plate heating temperatures. The effects of printing temperatures and plate heating temperatures on the estimated W_f_, D_f_ and T_DSI_ are plotted in Figure 17.

Figure 17a,b show that estimated fused contact width and depth of rubbery region can be improved by either increasing the printing temperature, increasing plate temperature, or both. A full width of bead is predicted to be fused to the ABS substrate when printing temperature of 250 °C and plate heating temperature of 50 °C is utilized. From the cross-section of the actual bead in Figure 6, the actual width of fused contact is approximated to be 75% of the width of the bead. Hence, a 75% width of bead, or 0.30 mm in this paper, is desired for a proper bonding formation between PLA materials and ABS substrate. This can be achieved by applying plate heating with a temperature of 50 °C during the deposition process at printing temperatures ranging between 230 °C to 250 °C. The deposition process using a combination of printing temperature of 250 °C and plate heating temperature of 40 °C can satisfy the desired minimum fused contact width of 0.30 mm. These four combinations of printing temperatures and plate heating temperatures are suggested for proper depositing PLA materials on ABS substrate. The proposed plate heating temperature can be achieved by implementing a localized pre-deposition heating approach on the substrate studied in [39], which can be applicable for both simple and complex-shaped parts. From the graph in Figure 17a, a proper process map for depositing PLA materials on ABS substrate is obtained.

The depths of the rubbery region formed by using these four suggested combinations of printing temperatures and plate heating temperatures are found to be greater than 25% of the height of the bead, as shown in Figure 17b. The lowest T_DSI_ among these four suggested combinations is 125.86 °C. This T_DSI_ is nearly 16 °C higher than the glass transition temperature of ABS. This information is useful for the selection of deposition process parameters during the deposition of PLA materials on ABS substrate.

## 4. Conclusions

In this paper, finite element analyses were implemented to investigate the deposition characteristics of PLA material on an ABS substrate during a ME process. Control of printing temperature during a ME process is vital to determine proper deposition of plastic on another dissimilar plastic substrate due to differences in melting temperatures of dissimilar plastics. A steady state heat transfer FE model of extruder unit was proposed and validated to investigate the effects of printing temperature and extruded length of PLA filament on temperature distributions. By comparing with experimental data during an ME deposition, the actual deposition temperature on deposit–substrate interface can be predicted using estimated temperature at end of extruded material. The result of FEAs provided sufficient information for the selection of 0.2 mm stand-off distance from the viewpoint of estimated temperature at end of extruded material.

A moving heat source and element activation algorithm was adapted from a DED process to simulate a ME process during single bead deposition of PLA on an ABS plate. Transient heat transfer FEAs were applied to reveal the deposition characteristics throughout the length of bead deposition. The proposed FE model for the deposition process successfully estimates the heating characteristics and peak temperatures at the time PLA material is deposited on the substrate. The FE model was validated through comparisons between actual temperature measurement values and estimated temperature histories.

Plate heating is considered to improve the fusion contact area between ABS and PLA. The influences of plate heating temperature and printing temperature on the formation of the rubbery region in the ABS substrate during deposition were studied using the validated FE model. Good bonding formation was estimated based on the estimated fused contact width and glass transition temperature of ABS. Subsequently, it was revealed that the ME process with combinations of printing temperatures and plate heating temperatures, which results in deposition temperature T_DSI_ greater than 125.86 °C, would provide a desirable bonding formation between PLA material and ABS substrate. The presented numerical investigations can be extended to study proper ME process parameters for different combinations of thermoplastics. The proposed plate heating temperature can be applied for complex-shaped parts by implementing a localized pre-deposition heating approach studied in [39].

## Figures and Tables

**Table 1:** Parameters for finite element model during the extrusion process.

Printing Temperature (T_p_, °C)	Extruded Length of PLA from the End of Nozzle Orifice (L_e_, mm)
230, 240, 250	0.1, 0.2, 0.3, 0.4, 0.5

**Table 2:** Parameters for finite element model during deposition process.

Printing Temperature (T_p_, °C)	ABS Substrate Initial Temperature (T_s_, °C)	Deposition Speed (V, mm/s)	Height of Bead (h_b_, mm)
230, 240, 250	20	10	0.2

**Table 3:** Parameters for finite element model during the deposition process.

Penetration Depth (h_b_, mm)	Effective Heat Radius at Surface of Bead (r_e_, mm)	Effective Heat Radius at *h_b_* (*r_i_*, mm)
0.2	0.2	0.199

**Table 4:** Calibrated efficiency for heat flux model.

Printing Temperature (T_p_, °C)	Calibrated Efficiency (*η*, %)
230, 240, 250	100

**Table 5:** Parameters for finite element model to investigate deposition process of PLA on ABS with plate heating.

Printing Temperature (T_p_, °C)	ABS Substrate Heating Temperature (T_s_, °C)	Deposition Speed (V, mm/s)	Height of Bead (h_b_, mm)
230, 240, 250	30, 40, 50	10	0.2

**Table 6:** Comparison of measured temperatures and estimated temperatures at the nozzle for different printing temperatures.

Printing Temperature (T_p_, °C)	Measured Temperature at Nozzle (T_n,actual_, °C)	Estimated Temperature at Nozzle (T_n,est_, °C)
230	215.0	213.0
240	223.2	222.4
250	233.4	233.1

**Table 7:** Comparison of mean peak temperature of thermocouples and estimated temperature at end of filament for L_e_ = 0.2 mm using different printing temperatures.

Printing Temperature (T_p_, °C)	Mean Peak Temperature of TC05, TC15, TC25, and TC35 (T_deposit_, °C)	Estimated Temperature at End of Extruded Material for L_e_ = 0.2 mm (T_e_, °C)
230	108.18	104.6
240	109.98	108.6
250	116.72	112.9

## Data Availability

Data sharing is not applicable to this article.

## Abbreviations

ME	Material Extrusion
FDM	Fused Deposition Modelling
ABS	Acrylonitrile Butadiene Styrene
PLA	Polylactic Acid
mFFF	Metallic Fused Filament Fabrication
SLA	Stereolithography
FE	Finite Element
FEA	Finite Element Analysis
DED	Directed Energy Deposition
DSI	Deposit–Substrate Interface
